# The Cushing Reflex: Oliguria as a Reflection of an Elevated Intracranial Pressure

**DOI:** 10.1155/2017/2582509

**Published:** 2017-05-15

**Authors:** K. Leyssens, T. Mortelmans, T. Menovsky, D. Abramowicz, Marcel Th. B. Twickler, L. Van Gaal

**Affiliations:** ^1^Department of Internal Medicine, University of Antwerp, Antwerp, Belgium; ^2^Faculty of Medicine, University of Antwerp, Antwerp, Belgium; ^3^Department of Neurosurgery, Antwerp University Hospital, Edegem, Antwerp, Belgium; ^4^Department of Nephrology, Antwerp University Hospital, Edegem, Antwerp, Belgium; ^5^Department of Endocrinology, Diabetology and Metabolism, Antwerp University Hospital, Edegem, Antwerp, Belgium

## Abstract

Oliguria is one of the clinical hallmarks of renal failure. The broad differential diagnosis is well known, but a rare cause of oliguria is intracranial hypertension (ICH). The actual knowledge to explain this relationship is scarce. Almost all literature is about animals where authors describe the Cushing reflex in response to ICH. We hypothesize that the Cushing reflex is translated towards the sympathetic nervous system and renin-angiotensin-aldosterone system with a subsequent reduction in medullary blood flow and oliguria. Recently, we were confronted with a patient who had complicated pituitary surgery and displayed multiple times an oliguria while he developed ICH.

## 1. Introduction

Oliguria is one of the clinical hallmarks of renal failure, but a rare cause of oliguria is intracranial hypertension. Recently, we were confronted with a patient who had complicated pituitary surgery and displayed multiple times an oliguria, while he developed intracranial hypertension.

## 2. Case Report

A 37-year-old male presented with a progressive hemiparesis due to a pituitary macroadenoma. He obtained significant tumor debulking, resulting in a panhypopituitarism including diabetes insipidus for which he was treated with desmopressin in a fixed daily dosage scheme and a strict control of intravenous/enteral fluids. He had stable plasma sodium levels (ranging within 138–150 mmol/L) and urine output (ranging within 1000–3540 ml/day).

At the ward, he suddenly developed oliguria with subsequent reduced consciousness. Clinically, he was in an euvolemic state. Desmopressin was discontinued, but oliguria persisted. His daily fluid intake was not changed. No perturbations were observed in kidney function including electrolytes. A renal ultrasound showed no signs of ureter obstruction.

**Figure 1:**
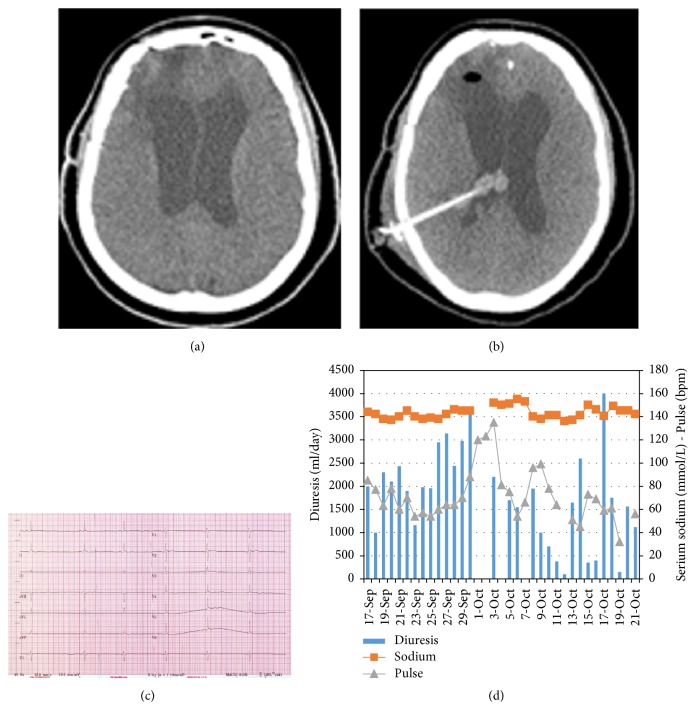
Oliguria during intracranial hypertension. (a) Head CT scan, during the first episode of oliguria, shows an obstructive hydrocephalus. (b) Head CT scan, during the second episode of oliguria, shows a hydrocephalus due to obstructed VP-shunt due to intraventricular bleeding. (c) ECG with sinus bradycardia (30 bpm) observed during the second episode of oliguria. (d) This graphic displays the patient's serum sodium levels (mmol/L) in comparison with diuresis and pulse. We notice two times a significant oliguric phase on 11-Oct and 19-Oct. These were the days that intracranial hypertension was present and surgery for decompression was executed. We see a significant decrease in diuresis on 15-16-Oct; this was due to excessive high doses of desmopressin (8 *µ*g iv).

Intracranial hypertension was suspected and a head CT scan showed hydrocephalus with cerebral edema (Figure 1(a)). After a 48-hour period of oliguria, a ventriculoperitoneal (VP) shunt was placed and postoperatively his diuresis turned to normal limits (1650 ml/day) with regain of full consciousness.

One week later, our patient developed oliguria again. This time he was clinically hypervolemic, but with normal kidney function and electrolytes. Desmopressin was discontinued, but diuresis remained restricted. In this time interval, the patient was no longer on a fixed schedule of desmopressin (ranging between 8 *µ*g intravenously and 10 *µ*g intranasally). During that day, consciousness decreased with significant bradycardia in parallel (30 bpm; Figure 1(c)). A new head CT scan showed progressive hydrocephalus due to intraventricular bleeding (Figure 1(b)). The blocked shunt was removed and an extracranial drain was inserted. Our patient's clinical condition recovered, with a polyuric phase after decompression treated with desmopressin.

During the two events of oliguria, hydrocortisone substitution was increased in line with recent clinical guidelines.

## 3. Discussion

Oliguria mostly reflects the kidney response to a decrease in circulating blood volume. Except in our case, we believe that it served as an alarming sign for increasing intracranial pressure (ICP) and clinical deterioration. The relationship between oliguria and intracranial hypertension (ICH) can be explained by the Cushing reflex, which is a hypothalamic response to ischemia and exists of a triad, hypertension, bradycardia, and apnea [1]. It triggers an increase in sympathetic outflow to the heart, kidney, and vessels as an attempt to increase arterial blood pressure and total peripheral resistance in order to maintain cerebral perfusion pressure and cerebral blood flow during ICH. Translated on the kidney level, the sympathetic nervous system will promote the release of renin with subsequent activation of the renin-angiotensin-aldosterone system. If concentrations of angiotensin II and antidiuretic hormone are high, they can induce a renal afferent vasoconstriction and reduced medullary blood flow to such an extent so as to account for oliguria [2–5]. The increase in blood pressure will then stimulate baroreceptors in the carotids leading to an activation of the parasympathetic nervous system which slows down the heart rate, causing bradycardia.

This principle is already described in animal studies. James and Wise studied the effect of raised ICP on renal function in the dog and found a significant decrease in the urinary excretion of sodium, in glomerular filtration rate, and in renal plasma flow [4]. They explained their findings on the basis of a decreased blood flow to the kidney, secondary to peripheral vasoconstriction, including renal arteriolar constriction, which has been shown to be initiated by ICH. Kobrine et al. likewise described a study that demonstrated oliguria in dogs during periods of increased ICP accompanied by an elevated systemic arterial pressure [5]. Finally, Salk and Weinstein [2] confirmed these results in dogs and explained their finding of a decreased urinary flow rate on the basis of a renal vasoconstriction causing a decrease in the renal blood flow.

## 4. Conclusion

In our case, oliguria presented two times as a clinical warning sign for ICH. This clinical presentation is an intriguing example in humans of direct communication between brain and kidney, as already presented in various animal studies before. We hypothesize that Cushing reflex is translated towards the sympathetic nervous system and renin-angiotensin-aldosterone system with a subsequent reduction in medullary blood flow and oliguria.

## References

[1] Boron W. F., Boulpaep E. L. (2005). *Medical Physiology*.

[2] Salk M. R., Weinstein R. E. (1939). On the effects of acutely raised intracranial pressure on diuresis in the dog. *American Journal of Physiology*.

[3] Fassot C., Lambert G., Elghozi J.-L., Lambert E. (2001). Impact of the renin-angiotensin system on cerebral perfusion following subarachnoid haemorrhage in the rat. *Journal of Physiology*.

[4] James I. M., Wise B. L. (1969). The effect of raised intracranial pressure on the handling of sodium by the canine kidney. *Jounal of Clinical Science*.

[5] Kobrine A. I., Kempe L. G., Mullane J. F. (1973). Natriuresis in the rhesus monkey after intracranial hypertension. *Annals of Surgery*.

